# Host status of morning-glory (*Ipomoea* spp.) to *Meloidogyne* species

**DOI:** 10.21307/jofnem-2021-018

**Published:** 2021-02-15

**Authors:** Tiago Edu Kaspary, Ismail Teodoro de Souza Júnior, Rodrigo Ferraz Ramos, Cristiano Bellé

**Affiliations:** 1 Instituto Nacional de Investigación Agropecuaria (INIA) La Estanzuela, Colonia, Uruguay; 2 Universidade Federal de Pelotas, Pelotas, Rio Grande do Sul, Brazil; 3 Universidade Federal de Santa Maria, Santa Maria, Rio Grande do Sul, Brazil; 4 Phytus Group, Estação experimental de Itaara, 97185-000, Itaara, Rio Grande do Sul, Brazil

**Keywords:** Weeds, Root-knot nematode, *Ipomoea* spp., Reproduction, Susceptibility

## Abstract

Weeds can be hosting and alternative multipliers of root-knot nematodes (*Meloidogyne* spp.). Among the main weeds, species of the genus *Ipomoea* stands out for their cosmopolitan presence and the negative impact on crops. In addition, they can behave as hosts and promote the reproduction of pests, diseases, and nematodes. However, the ability of Meloidogyne nematodes to infect morning-glory (*Ipomoea* spp.) is little understood. In this context, the objective was to evaluate the reproduction of *M. arenaria*, *M. enterolobii*, *M. ethiopica*, *M. hapla*, *M. incognita*, *M. javanica*, *M. luci*, and *M. morocciensis* in *I. grandifolia*, *I. hederifolia*, *I. nil*, *I. purpurea*, and *I. quamoclit.* Plants were individually inoculated with 5,000 eggs and second-stage juveniles and kept in a greenhouse for 60 days. The design was completely randomized with six repetitions. After this period, the root system of each plant was evaluated to gall index (IG) and reproduction factor (RF). It was verified that the eight species of *Melodoigyne* have the capacity to parasitize *I. grandifolia*, *I. hederifolia*, *I. nil*, *I. purpurea*, and *I. quamoclit*, showing the susceptibility of these weeds to the plant-parasitic nematodes. The highest RF were observed for *M. enterolobii* with values of 12.5 and 12.9 for *I. quamoclit* and *I. hederifolia*, respectively. While *M. arenaria* obtained the lowest values, with RF ≤ 4.0 for all species of *Ipomoea*. Thus, weed species of the *Ipomoea* genus are potential hosts and multipliers of root-knot nematodes, making it important to be considered in integrated management strategies for these plant-parasitic nematodes.

Weeds are an important biotic factor that limits agricultural crops. The damage caused to the development and productivity of crops can be generated by direct competition for resources, such as water, light, and nutrients, or by the action of allelochemicals produced by weeds (Ramesh et al., 2017). In addition, the presence of weeds can compromise the final quality of agricultural products, by contamination with their vegetable remains and seeds, or by becoming alternative hosts of pests and diseases, multiplying these and becoming a source of inoculum for future infestations (Anwar et al., 2009; Bellé et al., 2017b; Singh et al., 2010).

Currently, among the most problematic weeds for agriculture, the genus *Ipomoea* stands out, popularly known as morning-glory. Among the main species of this genus, some are of agricultural importance in annual and perennial crops and are reported as weeds in 40 crops in more than 40 countries (Holm et al., 1997; Chauhan and Abugho, 2012), especially *Ipomoea hederifolia* L.*, Ipomoea quamoclit* L.*, Ipomoea purpurea* L.*, Ipomoea grandifolia* (Dammer) O’Donell and *Ipomoea nil* (L.) Roth (Kissmann and Groth, 1999). The main characteristic of these plants are the volatile stems and branches, which give rise to the climbing habit (Lorenzi, 2000). In addition to competing with culture, these plants can interfere with cultural practices, especially mechanized harvesting, whose operational efficiency of the harvester is reduced by the fact that the plants are wrapped around the culms of the crop (Elmore et al., 1990; Lorenzi, 2000). These factors, combined with a wide geographical distribution, high production capacity and propagation of propagules and the difficulty in handling amplify the negative effect of weeds on agriculture.

The knowledge of weeds that act as alternative hosts for pests and diseases have been used as an integrated management tool in several agricultural crops. In this context, several weed species have been reported as hosts of plant-parasitic nematodes, among which the root-knot nematodes (*Meloidogyne* spp. Göldi). This phytoparasite genus has the greatest impact on crops in the world, in addition to being the genus most frequently found in parasitic weed roots (Bellé et al., 2019; Ferraz et al., 1978; Moens and Perry, 2009). In Brazil, an increasing number of studies have been developed that report weeds, present in agricultural areas, as natural hosts of several species of nematodes of the genus *Meloidogyne* (Bellé et al., 2016; Groth et al., 2017; Kaspary et al., 2017).

Management options for root-knot nematodes are limited, due to their wide range of hosts across various cultures and the lack of effective and environmentally acceptable control methods (McSorley, 1992; Ritzinger and Fancelli, 2006; Starr et al., 2007). There are few cultivars with resistance to root-knot nematodes, and the existing ones are restricted to one or two species of this nematode (Sikora et al., 2018). Due to plant and nematicide resistance limitations, crop rotation is the nematode control method widely used by producers in different parts of the world (Bellé et al., 2020; Ramos et al., 2019). However, the effectiveness of crop rotation for the management of root-knot nematodes is drastically reduced due to the presence of weeds in crops and during the off-season (Bellé et al., 2016; Koenning et al., 1999).

The root-knot nematode*s M. javanica* (Treub) Chitwood, *M. incognita* (Kofoid and White) Chitwood, *M. arenaria* (Neal) Chitwood and *M. enterolobii* Uang and Eisenback are distributed in the main areas of agricultural production in Brazil. *M. hapla* Chitwood*, M. morocciensis* Rammah and Hirschmann. *M. luci* Carneiro et al. and *M. ethiopica* Whitehead, on the other hand, are milder climate species, being more limited to these regions. Some of these are of special interest (*M. enterolobii* and *M. ethiopica*) due to their ability to overcome some root-knot nematode resistance genes (Carneiro et al., 2016; Galbieri et al., 2020). Currently, little is known about the reproduction of root-knot nematodes in species of morning-glory (*Ipomoea* spp.) in Brazil. In this context, this study aimed to evaluate the reproduction of eight species of *Meloidogyne* in *Ipomoea grandifolia*, *I. hederifolia*, *I. nil*, *I. purpurea*, *I. quamoclit.*

The experiment was conducted a greenhouse, in independent trials from November 2018 and February 2019, where the reaction of five species of morning-glory (*I. grandifolia, I. hederifolia, I. nil, I. purpurea*, and *I. quamoclit*) to eight *Meloidogyne* species, in a greenhouse a 25°C±3°C. Weeds were identified and classified according to Ferreira and Miotto (2009). A completely randomized experimental design was used with six replications.

It was used with a substrate for the experiment a mixture of soil (Rhodic Ferralsol – WRB 2015) and sand, at proportion of 1:2, with autoclave disinfection before use. The soil was composed of clay=48%; water pH value=6.4; Shoemaker MacLean, and Pratt (SMP) test=6.6; organic matter=3.3%; P=10.7 mg dm^−3^; K=86 mg dm^−3^; Ca=5.2 cmolc dm^−3^; Mg=4.9 cmolc dm^−3^; and S=8.5 cmolc dm^−3^. In total, 15 days after emergence of morning-glory on plant were transplanted in each put containing 2,000 dm^−3^ the substrate.

As inoculum of the root-knot nematode, pure populations of *M. arenaria* (Est A2)*, M. enterolobii* (Est M2)*, M. ethiopica* (Est E3)*, M. hapla* (Est H1)*, M. incognita* (Est I2)*, M. javanica* (Est J3)*, M. luci* (Est L3), and *M. morocciensis* (Est A3) confirmed using the electrophoresis technique for isoenzymes, as proposed by Carneiro and Almeida (2001), were maintained and multiplied in ‘Santa Cruz’ tomato plants (*Solanum licopersycum* L.). The nematode inoculum was obtained from the root system of tomato plants kept in a greenhouse, using the method of Hussey and Barker (1973), extracted with 0.5% NaOCl, using a blender instead of manual shaking. The experimented plants were inoculated five days after transplantation with 3 ml. of suspension containing 5,000 eggs and second juvenile (J2) per plant in three holes approximately two cm deep around the plant. ‘Santa Cruz’ tomatoes were used as a control of the viability of the inoculum of the nematode population.

The roots of each morning-glory plant were washed individually 60 days after inoculation. The galls were counted following the methodology proposed by Taylor and Sasser (1978), where 0=no galls, 1=1 to 2, 2=3 to 10, 3=11 to 30, 4=31 to 100, and 5=more than 100 galls per root system. Subsequently, the root systems were processed following the method of Hussey and Barker (1973), to obtain the final number of nematodes. From this, the reproduction factor (final population/initial population) was determined, as described by Oostenbrink (1966). Thus, each morning-glory species was classified as RF=0 for immune, RF <1 for resistant, and RF>1 was susceptible for each nematode species.

The similarity form, the experiment was performed twice times and compared by preliminary analysis of variance (ANOVA). This analysis was not significant for first and second round, allowing the use of the data together. Subsequently, the treatments were compared by Scott-Knott clustering test at probability level of 5%, using the GENES software (Cruz, 2006).

The results obtained demonstrate a similar behavior of the *Ipomoea* species evaluated in relation to the reaction to the different *Meloidogyne* species. The viability of the inoculum was confirmed by the reproduction factor above 17.5 found in *S. lycopersicum*, used as a control, for all nematode species evaluated (Table 1). The gall indexes presented by the five *Ipomoea* species were greater than 4.5 when they were subjected to the eight species of root-knot nematode (Table 1).

**Table 1.** Galls index (GI), and factor of reproduction (RF) of *M. arenaria*, *M. enterolobii*, *M. ethiopica*, *M. hapla*, *M. incognita*, *M. javanica*, *M. luci*, and *M. morocciensis* in *I. grandifolia*, *I. hederifolia*, *I. nil*, *I. purpurea*, and *I. quamoclit*.

Species	*M. arenaria*	*M. enterolobii*	*M. ethiopica*	*M. hapla*	*M. incognita*	*M. javanica*	*M. luci*	*M. morocciensis*
*Galls index* ^*a*^								
*I. grandifolia*	5.0 ns	5.0 ns	5.0 ns	5.0 ns	5.0 ns	5.0 ns	5.0 ns	5.0 ns
*I. hederifolia*	5.0	5.0	5.0	4.5	5.0	5.0	5.0	5.0
*I. nil*	4.5	5.0	5.0	5.0	5.0	5.0	5.0	5.0
*I. purpurea*	4.5	5.0	5.0	5.0	5.0	5.0	5.0	5.0
*I. quamoclit*	5.0	5.0	5.0	5.0	5.0	5.0	4.5	5.0
*S. licopersycum*	5.0	5.0	5.0	5.0	5.0	5.0	5.0	5.0
*Reproduction factor* ^*c*^								
*I. grandifolia*	2.7 B c	10.4 B a	8.6 A a	5.5 A c	6.8 B b	9.7 A a^c^	8.5 A a	6.1 A b
*I. hederifolia*	3.4 A c	12.5 A a	10.1 A a	3.0 B c	9.0 A a	6.1 B b	9.1 A a	6.5 A b
*I. nil*	3.3 B c	9.8 B a	7.1 B b	5.2 A b	5.7 B b	7.8 B b	9.3 A a	4.9 B b
*I. purpurea*	3.8 A c	11.0 B a	7.6 B b	4.7 A c	7.8 A b	8.9 A b	6.7 B b	5.5 A c
*I. quamoclit*	4.0 A c	12.9 A a	9.7 A b	3.2 B c	8.9 A b	6.7 B b	3.6 C c	4.5 B c
*S. licopersycum*	28.8	35.68	25.57	19.4	21.6	24.5	36.7	45.5
CV (%)	25.56							

**Notes:** ns=not significant. ^a^Galls Index based on Taylor and Sasser (1978): 0=no galls; 1=1 to 2; 2=3 to 10; 3=11 to 30; 4=31 to 100, and 5=more than 100 galls per root system; ^b^reproduction factor=final population (PF)/initial population (Pi=5,000); ^c^means followed by the same uppercase letter in a row and lowercase in a column, not differ significantly by the Scott-Knott test at 5% probability.

The species *I. grandifolia* and *I. purpurea* presented the largest reproduction factors for *M. javanica*, with an RF of 9.7 and 8.9, respectively (Table 1). While for *M. incognita*, the highest index was observed in *I. hederifolia* (RF=9.0), whereas *I. nil* obtained the lowest reproduction factor for this nematode (RF=5.7). *M. arenaria* showed greater capacity to multiply in plants of the species *I. quamoclit, I. purpurea* and *I. hederifolia* with RFs greater than 2.7 on *I. grandifolia* (Table 1). Plants of the species *I. grandifolia, I. nil*, and *I. purpurea* were more likely to multiply the nematode *M. hapla* when compared to *I. hederifolia* (Table 1). For *M. ethiopica*, the RFs varied from 7.1 to 10.1, with the weeds *I. hederifolia, I. quamoclit*, and *I. grandifolia* having the highest RF (Table 1). Whereas the species *I. quamoclit* and *I. hederifolia* also resulted in the highest RF for the nematode *M. enterolobii,* with reproduction factors of 12.5 and 12.9, respectively (Table 1). For *M. luci*, the species *I. nil, I. hederifolia* and *I. grandifolia* presented the highest RF, varying from 8.5 to 9.3, while for *I. quamoclit*, the lowest reproduction factor was observed for this nematode (RF=3.6).

Species of *Ipomea* showed little variation in the reproductive capacity of *M. morocciensis*, however, *I. hederifolia, I. grandifolia* and *I. purpurea* showed the highest RF statistically, ranging from 5.5 to 6.5 (Table 1). In contrast, *I. nil*, was the species that presented the lowest RF values for most (6) of the *Meloidogyne* species evaluated, while *I. hederifolia* was the one that presented the highest RF values for six of the eight *Meloidogyne* species evaluated.

From the RF averages of the different plant-parasitic nematodes facing the different species of *Ipomoea*, it was found that *M. enterolobii* presenting the highest reproduction capacity with RF of 11.32 (Fig. 1). This value is about 70 and 62% higher than values found for *M. arenaria* and *M. hapla*, with an average RF of 3.44 and 4.32, respectively. For the other species, the mean RF varied from 4.42 to 8.62, values considered high, demonstrating to which species of the genus *Ipomea* are excellent multipliers of the root-knot nematodes (Fig. 1).

**Figure 1:**
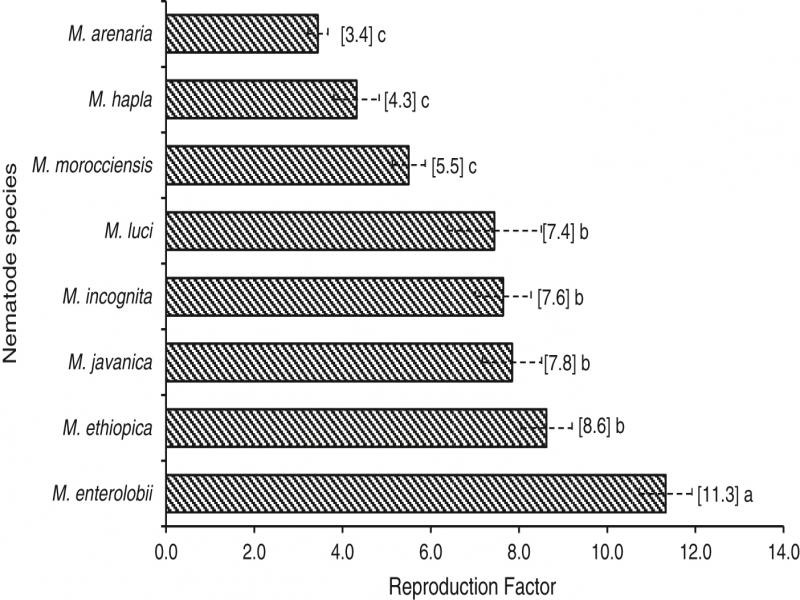
Average reproduction factor of *Meloidogyne* species, multiplied in *I. grandifolia, I. hederifolia, I. nil, I. purpurea,* and *I. quamoclit*. *Means followed by the same letter in the columns do not differ by the Scott-Knott test at 5% probability.

The high capacity to host and multiply the root-knot nematode species by the five *Ipomoea* species in the present study indicates that these weeds behaved as susceptible to these phytoparasites, the presence of which is the guarantee of the perpetuation of the plant-parasitic nematodes in cultivation areas (Bellé et al., 2017b; Singh et al., 2013). In addition, species of the genus *Ipomoea* have different germination flows as an aggravating factor throughout the year depending on temperature and humidity (Azania et al., 2009; Carvalho et al., 2008). This factor enhances the maintenance and reproduction of populations of different root-knot nematodes during the year.

The polyphagous capacity presented by the root-knot nematodes, care must be taken with the choice of the cultures to be implanted and the species in succession, whether commercial culture or not, in order to minimize the damage caused by *M. arenaria, M. enterolobii, M. ethiopica, M. hapla, M. incognita, M. javanica, M. luci and M. morocciensis.* Even in this context, weed control is of even greater importance, due to the proven and high susceptibility and potential host to the nematode species under study, demonstrating the polyphagous action of the pathogen, which can have negative consequences for the cultivation areas where they are located, mainly in areas with soybeans, beans, sugarcane, tobacco, vegetables, and fruit, where plant-parasitic nematodes are already widespread (Bellé et al., 2017b).

Considering the management of plant-parasitic nematodes, a way to control population growth population during the period of presence of cultivation of economic interest, is the proper management of weeds throughout the year (Bellé et al., 2016). The control method most used and considered most efficient for the management of morning-glory is the chemical with the use of pre and post-emergence herbicides, getting satisfactory control (Azania et al., 2009). In post-emergence, the use of herbicides is recommended only in the early stage of the morning-glory (2-4 leaves). Although to date, there are no reports of *Ipomoea* spp. resistant to different herbicides (Heap, 2020).

Thus, the difficulty in controlling weeds increases the management complexity of *Meloidogyne* spp. aggravating the harmful potential of the presence of these plants in crop fields. However, from the knowledge of the host range of *M. javanica*, *M. incognita*, *M. arenaria, M. hapla, M. ethiopica, M. enterolobii, M. luci*, and *M. morocciensis*, it will be possible to trace effective strategies in the management of these pathogens, reducing the damage caused to commercial crops. Finally, the reduction of agricultural losses caused by weeds and phytonematodes can be minimized with the integrated management of these two problems, which are interconnected and enhanced when they occur concurrently.
